# Multiwalled Carbon Nanotube for One-Step Cleanup of 21 Mycotoxins in Corn and Wheat Prior to Ultraperformance Liquid Chromatography–Tandem Mass Spectrometry Analysis

**DOI:** 10.3390/toxins10100409

**Published:** 2018-10-10

**Authors:** Dongmei Jiang, Dizhe Wei, Liuqing Wang, Shuai Ma, Yuanfang Du, Meng Wang

**Affiliations:** 1 Beijing Research Center for Agricultural Standards and Testing, No. 9 Middle Road of Shuguanghuayuan, Haidian District, Beijing 100097, China; jiangdm@brcast.org.cn (D.J.); weidz@brcast.org.cn (D.W.); Wanglq@brcast.org.cn (L.W.); Mas@brcast.org.cn (S.M.); Duyf@nercita.org.cn (Y.D.); 2 Risk Assessment Laboratory for Agro-Products, Ministry of Agriculture, No. 9 Middle Road of Shuguanghuayuan, Haidian District, Beijing 100097, China

**Keywords:** MWCNT, one-step cleanup, mycotoxin, UPLC–MS/MS, corn and wheat

## Abstract

One-step solid-phase extraction (SPE) using a multiwalled carbon nanotube (MWCNT) for simultaneous analysis of 21 mycotoxins, including nine trichothecenes, zearalenone (ZEN) and its derivatives, four aflatoxins, and two ochratoxins, in corn and wheat was developed. Several key parameters affecting the performance of the one-step SPE procedure—types of MWCNT, combinations with five sorbents (octadecylsilyl (C_18_), hydrophilic–lipophilic balance (HLB), mixed-mode cationic exchange (MCX), silica gel, and amino-propyl (NH_2_)), and filling amounts of the MWCNTs—were thoroughly investigated. The combination of 20 mg carboxylic MWCNT and 200 mg C_18_ was proven to be the most effective, allowing the quantification of all analyzed mycotoxins in corn and wheat. Under the optimized cleanup procedure prior to ultraperformance liquid chromatography–tandem mass spectrometry (UPLC–MS/MS) analysis, the method was validated by analyzing samples spiked at the limit of quantification (LOQ), two-times LOQ, and 10-times LOQ. Satisfactory linearity (*r*^2^ ≥ 0.9910), high sensitivity (LOQ in different ranges of 0.5–25 μg L^−1^), good recovery (75.6–110.3%), and acceptable precision (relative standard deviation (RSD), 0.3–10.7%) were obtained. The applicability of the method was further confirmed using raw samples of corn and wheat. In conclusion, the established method was rapid, simple and reliable for simultaneous analysis of 21 mycotoxins in corn and wheat.

## 1. Introduction

Corn and wheat are cereal grains widely consumed as food and feed and used as industrial raw materials worldwide. However, they are easily infected by spoilage fungi that cause the deterioration of grains, including discoloration, musty odors, tissue disintegration and loss of nutritional quality. Meanwhile, mycotoxins are produced under suitable temperature and humidity conditions, especially fusarium toxins, aflatoxins and ochratoxins, in corn and wheat [1,2]. Ingestion of these contaminated materials may be pathogenic in humans, because they may lead to serious health problems, such as liver, kidney or nervous system damage, immunosuppression, biphasic cellular response, and carcinogenesis [3,4]. Therefore, rapid, accurate and high-throughput analysis methods are needed to determine the global contamination level, assess the risk, and monitor the detoxification strategies of mycotoxins.

As we know, more than one mycotoxin may occur in cereal grains; for instance, deoxynivalenol (DON), zearalenone (ZEN) and nivalenol (NIV) co-occur in 74% of wheat samples [5], and at least eight fusarium toxins and ochratoxin A (OTA) were simultaneously detected in winter wheat [6]. Similarly, co-occurrence of OTA and ZEN was also reported in breakfast cereals [7]. As a result, it is an urgent problem to develop simultaneous analytical methods for multiclass mycotoxins. To date, numerous methods have been established to simultaneously analyze multiclass mycotoxins in different matrices by high-performance liquid chromatography–tandem mass spectrometry (HPLC–MS/MS) [8,9,10,11,12,13,14], but efficient pretreatment is still the main challenge. Due to the tedious process and matrix interference of quick, easy, cheap, effective, rugged and safe (QuEChERS) methods, poor stability and repeatability of liquid-phase microextraction (LPME), high cost of immunoaffinity chromatography (IAC), and poor selectivity of magnetic separation, solid-phase extraction (SPE) is still the main pretreatment method for analysis of multiclass mycotoxins [15,16].

For commercial SPE, silica gel, octadecylsilyl (C_18_), hydrophilic–lipophilic balance (HLB), and amino-propyl (NH_2_) can be obtained as adsorbent materials, but most of the commercial cartridges are not suitable for high-throughput screening for multiclass mycotoxins. Recently, multiwalled carbon nanotubes (MWCNTs), a new type of SPE absorbent, have been applied to sample pretreatment of mycotoxins due to their strong adsorption capacity, unique structure, and ease of being functionally modified as carboxylic MWCNT (MWCNT–COOH) and hydroxyl MWCNT (MWCNT–OH) [17,18]. As previously reported, MWCNTs have been used for the cleanup of type A trichothecenes and ZEN in cereals [19,20,21]. However, the procedure of sample pretreatment, including washing and eluting, is not convenient and also consumes significant time, especially when applied to analyze a great numbers of samples.

The purpose of our study is to establish a rapid, simple, sensitive and reliable procedure on the basis of one-step SPE cleanup using MWCNTs as sorbents followed by UPLC–MS/MS analysis, to simultaneously determine 21 mycotoxins in corn and wheat, including nine trichothecenes, ZEN and its derivatives, four aflatoxins, and two ochratoxins. The most important advantage of this method is that the cleanup is simple and fast, using a homemade MWCNT–COOH +C_18_ SPE cartridge as a filter, and the matrix effects are significantly reduced. This simple and rapid method can be used to simultaneously determine the targeted mycotoxins in corn and wheat with excellent efficiency.

## 2. Results and Discussion

### 2.1. UPLC–MS/MS

CORTECS C_18_ (1.6 μm, 2.1 × 100 mm, Waters, Milford, MA, USA) was employed as the separation column, and sharp chromatographic peaks, which means high sensitivity for each targeted mycotoxin in a spiked solvent sample at 10-times the limit of quantification (LOQ) level, are shown in Supplementary Figure S1. The results of MS/MS parameters demonstrated that ZEN and its derivatives, α-zearalenol (α-ZOL), β-ZOL, zearalanone (ZAN), α-zearalanol (α-ZAL) and β-ZAL, displayed more desirable results in negative electrospray ionization mode (ESI^−^), and the other 15 analytes showed much higher sensitivity in the positive mode of ESI (ESI^+^). The optimized parameters for each analyte—ionization mode, precursor and productions, collision energy and cone voltage—are shown in Supplementary Table S1.

### 2.2. Optimization of the SPE Cartridge

Effective sample pretreatment is critical for reducing matrix effects, especially trace amounts of mycotoxins existing in complex matrices [22]. MWCNTs can greatly reduce the matrix effect, owing to their strong adsorption capacity, and one can combine the features of MWCNTs with other adsorbent materials, thus forming a promising sorbent material for SPE in recent years. In our work, a simple and rapid homemade SPE procedure was developed to purify the prevalent multiclass mycotoxins, and also to minimize the matrix effects. Compared to the conventional SPE procedures, there was only one step, allowing the analytes to pass through and the co-extractive interference to be removed. Therefore, the SPE cartridge served as a rapid and convenient chemical filter. In this study, types of MWCNT, combinations with five sorbents and filling amounts were evaluated for optimization of the cleanup procedure. Matrix effects of the 21 analytes were investigated using corn, because it has a more complex matrix and more common mycotoxin contamination.

#### 2.2.1. Types of MWCNT

**Figure 1:**
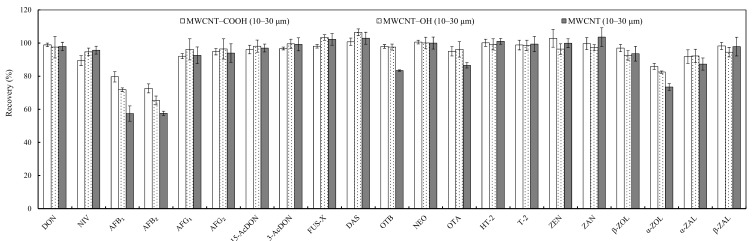
Effects of three MWCNT sorbents on recovery (%) of mycotoxins in corn. MWCNT, multiwalled carbon nanotube; MWCNT–COOH, carboxylic MWCNT; MWCNT–OH, hydroxyl MWCNT; DON, deoxynivalenol; NIV, nivalenol; AFB_1_, AFB_2_, AFG_1_, AFG_2_, aflatoxins; 15-AcDON, 15-acetyldeoxynivalenol; 3-AcDON, 3-acetyldeoxynivalenol; FUS-X, fusarenon X; DAS, diacetoxyscirpenol; OTA, ochratoxin A; OTB, ochratoxin B; T-2, T-2 toxin; HT-2, HT-2 toxin; NEO, neosolaniol; ZEN, zearalenone; α-ZOL, α-zearalenol; β-ZOL, β-zearalenol; ZAN, zearalanone; α-ZAL, α-zearalanol; β-ZAL, β-zearalanol. Vertical bar represents ± standard error (*n* = 3).

Initially, the effects of the type of MWCNT were investigated with 20 mg of the three multiwalled carbon nanotubes, MWCNT, MWCNT–COOH and MWCNT–OH, plus 200 mg silica gel. Compared to MWCNT–COOH, a relatively higher recovery of mycotoxins NIV, 3-acetyldeoxynivalenol (3-AcDON) and 15-acetyldeoxynivalenol (15-AcDON) was obtained when using unmodified MWCNT or MWCNT–OH as the sorbent, but the recovery of aflatoxins AFB_1_ and AFB_2_ was much lower, and even unacceptable (Figure 1). As a result, satisfactory recovery of 72.6–102.8% was observed using MWCNT–COOH as a sorbent for all the analytes. In addition, there was no significant difference in matrix effects for all mycotoxins in corn among the different types of MWCNT (Supplementary Figure S2); therefore, MWCNT–COOH was used for further study.

#### 2.2.2. Different Combinations of Sorbents

**Figure 2:**
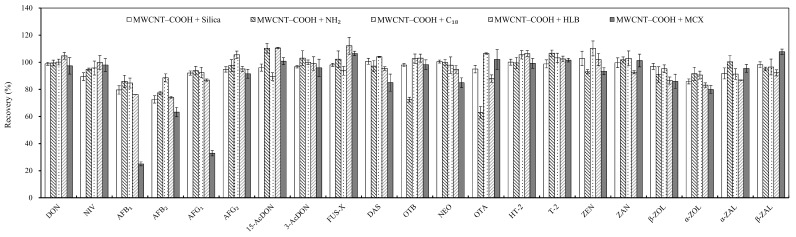
Effects of different solid-phase extraction (SPE) sorbents on the recovery (%) of mycotoxins in corn. For each of the five sorbents, 200 mg of the sorbent was well mixed with 20 mg of MWCNT–COOH and packed into the cartridge. NH_2_, aminopropyl; C_18_, octadecylsilyl; HLB, hydrophilic–lipophilic balance; MCX, mixed-mode cationic exchange. Vertical bar represents ± standard error (*n* = 3).

Different combinations of MWCNT–COOH with five sorbents, C_18_, HLB, NH_2_, mixed-mode cationic exchange (MCX) and silica gel, were used to investigate the cleanup efficiency of the 21 analytes. MCX is a common sorbent applied to both polar and nonpolar compounds [23]; however, recovery of MWCNT–COOH + MCX was significantly lower than that of other combinations, especially AFB_1_ (25.0%) and AFG_1_ (32.9%). NH_2_ is also a common sorbent that can remove similar types of compounds by interacting with chemicals, including some sugars, organic acids and pigments, through hydrogen bonding [24], but lower recovery of OTA (63.1%) was obtained here, and the recovery of OTB (72.4%) was much lower than those using other combinations; therefore, NH_2_ is not an eligible sorbent for the cleanup of ochratoxins (OT). Using silica gel and HLB achieved acceptable recovery for all the targeted mycotoxins; however, lower recovery of AFB_1_ and AFB_2_ was obtained compared to C_18_, which is commonly used for mycotoxin cleanup [25]. C_18_ achieved satisfactory recovery for all analytes, ranging from 84.7% to 110.2%, thus the combination of MWCNT–COOH and C_18_ was selected for further study (Figure 2).

#### 2.2.3. Amounts of MWCNT–COOH

**Figure 3:**
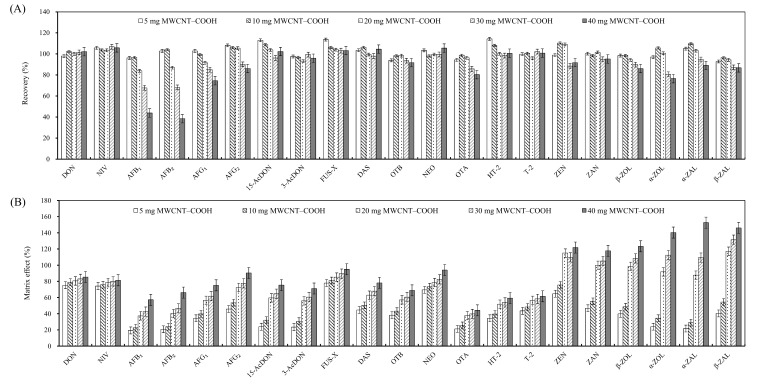
Effects of different amounts of MWCNT–COOH and 200 mg C_18_ on the recovery and matrix effect of mycotoxins in corn. Vertical bar represents ± standard error (*n* = 3). (**A**) Recovery (%); (**B**) Matrix effect (%).

The amount of absorbent was an important factor in the purification of targeted compounds, and in our study, different amounts of MWCNT–COOH (5, 10, 20, 30 and 40 mg) were applied to evaluate the cleanup efficiency of all the analytes, so as to achieve the highest recovery with the lowest amount of sorbent. As shown in Figure 3, the results indicated that there was no significant difference in recovery for the target mycotoxins as the amount of MWCNT–COOH increased from 5 mg to 20 mg; however, a significant decrease in the recovery of some analytes (aflatoxins, ochratoxins and zearalenones) was observed from 20 mg to 40 mg of MWCNT–COOH, although the matrix effects were dramatically removed by the increasing amounts due to the strong absorption of MWCNT–COOH (Figure 3). As a result, the combination of 20 mg MWCNT–COOH and 200 mg C_18_ was proven to be the most effective cleanup, and satisfactory recovery ranging from 84.0% to 109.1% was achieved for all analyzed mycotoxins in corn. In previous reports, the amount of MWCNT was always a key parameter to be optimized, and 100 mg of MWCNT was chosen to achieve complete adsorption in the dispersive or magnetic solid-phase extraction [20,26]. Here, only 20 mg of MWCNT–COOH was used to obtain the best results at lower cost, and meanwhile, the potential health hazard from exposure to MWCNT was also a consideration [27].

### 2.3. Evaluation of the Homemade One-Step Cleanup Method

**Figure 4:**
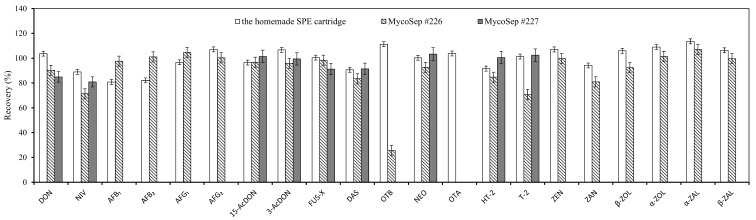
Efficiency comparison among the homemade SPE cartridge, MycoSep #226 column (Romer Labs Inc., Union, MO, USA), and MycoSep #227 column (Romer) on the recovery (%) of mycotoxins in corn. Vertical bar represents ± standard error (*n* = 3).

A comparison between homemade SPE and commercial multifunction cleanup (MFC) columns was performed using spiked samples (five-times LOQ) to evaluate the purification efficiency of the established one-step cleanup method. MycoSep #227 MFC columns are frequently utilized for purification of trichothecenes [28,29,30], and this was further supported by the present study based on the results that the recovery of trichothecenes with MycoSep #227 purification was 80.9–103.3% (Figure 4). MycoSep #226 MFC columns are also frequently applied to clean up aflatoxins and ZENs [31,32], and good recovery of aflatoxins and ZEN and its derivatives was also observed in our study (Figure 4); however, the above commercial MFCs could not be applied to the high-throughput determination of all the selected mycotoxins in cereal regulated by China, especially for OTA. According to our results, satisfactory recovery of all 21 mycotoxins, ranging from 80.8% to 113.6%, was obtained by our homemade SPE cartridge, suggesting that our SPE appeared to be a good choice for the simultaneous cleanup of prevalent mycotoxins in cereals with excellent performance and at much lower cost.

### 2.4. Method Validation

Method validation was performed in terms of LOQ, linearity, accuracy and precision for the 21 targeted mycotoxins in the selected model cereals, including corn and wheat, which are important cereals in northern China and easily exposed to mycotoxin contamination.

Calculated LOQs were set to 0.5–25 μg L^−1^ for the targeted mycotoxins with signal-to-noise ratios of 10 or more. As shown in Table 1, eight targeted mycotoxins, including the four AFs, OTA, OTB, ZEN and T-2, were quantified at 0.5 μg L^−1^, neosolaniol (NEO) and NIV were quantified at 25 μg L^−1^ because of low signal responses in the matrix, and the other mycotoxins were quantified at 10 μg L^−1^. Matrix-matched standard calibration of 21 analytes showed good linear relationships with correlation coefficients ≥0.9910 over the range of LOQ to 200 LOQ in corn and wheat.

Accuracy and precision of the method were evaluated by means of recovery and relative standard deviation (RSD) with five replicates at three levels: LOQ, two-times LOQ, and 10-times LOQ. The experimental data of recovery and RSD for each mycotoxin are summarized in Table 1. Overall, acceptable recovery ranging from 75.6% to 110.3% was obtained, and the intra- and interday RSDs of the 21 targeted mycotoxins were in the satisfactory range of 0.3–8.2% and 1.8–10.7%, respectively (Table 1), which indicated that this method had good repeatability and reproducibility.

All the validation data showed that the analytical method here was accurate and repeatable and could be applied to simultaneously analyze the 21 analytes in corn and wheat. So far as we know, limited information is available on cleanup of all major mycotoxins found in cereals using MWCNTs as sorbent. According to our results, a one-step cleanup procedure using MWCNT–COOH +C_18_ as a sorbent of the SPE cartridge was developed, which is feasible and efficient, and greatly simplified the sample pretreatment of 21 mycotoxins in cereals.

### 2.5. Method Application

As shown in Table 2, AFB_1_ was found in 19 corn samples at concentrations of 0.5–26 μg kg^−1^. ZEN was found in all corn samples at concentrations of 27.5–525 μg kg^−1^ and 13 wheat samples at concentrations of 26.8–602 μg kg^−1^. DON was found in seven corn samples and 27 wheat samples at higher concentrations. 3-AcDON and 15-AcDON were found in 11 and 12 wheat samples, respectively. The other 15 mycotoxins were not detected from the collected samples.

## 3. Conclusions

A novel and rapid one-step cleanup procedure using MWCNT–COOH +C_18_ as SPE sorbent was developed for purification of 21 mycotoxins in corn and wheat. In comparison with the conventional SPE method, this one-step cleanup method shortened the time for sample pretreatment with good recovery and minimized matrix effects. The homemade SPE cartridge also greatly reduced cost compared with the expensive immune-affinity column and various MycoSep multifunctional cleanup columns. The combination of the one-step cleanup procedure with UPLC–MS/MS analysis provided a rapid, sensitive and reliable method to simultaneously determine the 21 targeted mycotoxins, which was validated in corn and wheat, suggesting an application of the proposed method in the analysis of the targeted mycotoxins in various crops.

## 4. Material and Methods

### 4.1. Reagents

MS-grade acetonitrile and methanol were purchased from Thermo Fisher Scientific (Fair Lawn, NJ, USA). Ammonium acetate (NH_4_Ac), acetic acid and citric acid were purchased from Sigma-Aldrich (Buchs, Switzerland). Milli-Q quality water was obtained from Millipore (Billerica, MA, USA). Sorbents of C_18_, HLB, NH_2_, MCX and silica gel were purchased from Beijing Solarbio Science and Technology Company Limited (Beijing, China). MWCNT adsorbent materials of 10–30 μm length (8 nm i.d., 500 m^2^ g^−1^) were obtained from Nanjing XFNano Materials Technology Company Limited (Nanjing, China).

### 4.2. Standards

Certified standards of 4 aflatoxins (AFs: AFB_1_, AFB_2_, AFG_1_, AFG_2_), OTA, OTB, DAS, NIV, NEO, T-2, HT-2, DON, 3-AcDON, 15-AcDON, ZEN, α-ZOL, β-ZOL and fusarenon X (FUS-X) were obtained from Romer Labs Inc. (Union, MO, USA). ZAN and α-ZAL were purchased from Sigma-Aldrich (Buchs, Switzerland), and β-ZAL was purchased from Pribolab Private Limited (Immunos, Singapore).

The individual stock solutions of AFs, ZEN, T-2, OTA and OTB were dissolved in acetonitrile at 10 mg L^−1^ and the other 13 standards were prepared in acetonitrile at 100 mg L^−1^. AFs, ZEN, T-2, OTA and OTB were prepared in enough acetonitrile at 0.5 mg L^−1^ as an intermediate mixed solution (Mix A). A similar procedure was carried out for Mix B including DAS, DON, FUS-X, HT-2, 3-AcDON, 15-AcDON, ZAN, α-ZAL, β-ZAL, α-ZOL and β-ZOL toxins, at a stock concentration of 10 mg L^−1^. Mix C, of NEO and NIV at a concentration of 25 mg L^−1^, was obtained in the same way. Then, 1 mL of each mixture, Mix A, Mix B and Mix C, and 7 mL acetonitrile, were mixed to obtain 10 mL of total mixed stock of the standard solution, which was stored at −20 °C in the dark. Working solutions were freshly diluted with the blank matrix or acetonitrile before UPLC–MS/MS analysis.

### 4.3. Samples

Approximately 1 kg of corn and wheat samples, which were confirmed to be free of the 21 analytes, were purchased from local markets in Beijing, China. In total, 59 corn niblets and 53 wheat kernels were collected in Hebei Province, China, for method application. All the samples were ground into fine powder with a blender. Before extraction, these samples were stored at −20 °C.

### 4.4. Preparation of SPE Cartridges

Five SPE cartridges were obtained by placing MWCNT–COOH plus each of 5 sorbents (silica gel, NH_2_, HLB, MCX and C_18_) inside 6 mL polypropylene tubes with a frit at the 2 sides. For a combination of sorbents, 200 mg of each of the 5 sorbents was well mixed with 20 mg MWCNT–COOH and packed into the cartridge. Then, the cartridges were treated with 5 mL of acetonitrile and 5 mL of Milli-Q quality water.

### 4.5. Extraction

Mycotoxins were extracted according to a previous report [13] with minor revision. Briefly, 5.0 g of fine homogenized cereal was mixed with 4 mL water and 21 mL acetonitrile containing 100 mM citric acid. After being shaken for 30 min and centrifuged for 10 min at 10,000 rpm, the supernatant was passed through the homemade SPE cartridge, and 5 mL outflow was collected directly into a centrifuge tube. During this process, large matrix interferences were retained on the SPE column. The cleanup extract in the centrifuge tube was then evaporated to almost dryness at 50 °C under a gentle nitrogen stream, and the residue was dissolved in 1 mL acetonitrile/water (3:7 *v*/*v*). After being forced through a 0.22 μm polytetrafluoroethylene (PTFE) filter membrane (Pall, MI, USA), 5 μL of the final solution was analyzed by UPLC–MS/MS.

### 4.6. UPLC–MS/MS Conditions

UPLC–MS/MS conditions were optimized according to a previous report [13]. The UPLC system consisted of an Acquity UPLCTM from Waters (Milford, MA, USA). The analyte separation was carried out on a CORTECS^®^ UPLC C_18_ column with particle size of 1.6 μm (2.1 × 100 mm; Waters), and the column temperature was maintained at 40 °C. Then, 1 mM NH_4_Ac in Milli-Q quality water (solvent A) and methanol (solvent B) served as the mobile phases at a flow rate of 0.3 mL min^−1^ for 9 min with the following gradient elution: 0–0.5 min (95% A), 6.0 min (25% A), 7.5 min (10% A), 7.6–9.0 min (95% A). The samples were maintained at 10 °C in the instrument during all runs until injection, which volume was 5 μL.

The UPLC system was coupled with a Xevo TQ-S equipped with an ESI source (Waters, Milford, MA, USA). Mass spectrometry was performed via both positive and negative modes of electrospray ionization due to the different structural properties of the analytes. Optimized parameters for the mass spectrometry were as follows: capillary voltage, +2.5 kV/−0.8 kV; desolvation temperature, 500 °C; source temperature, 150 °C; desolvation gas flow, 1000 L h^−1^; and cone gas flow, 150 L h^−1^. The analytes were detected in multiple-reaction monitoring (MRM) mode and the optimized parameters for each analyte are shown in Supplementary Table S1. Data acquisition and processing were performed using MassLynx^TM^ 4.1 software (Waters, Milford, MA, USA).

### 4.7. Method Validation

The method was validated for sensitivity (LOQ), linearity, accuracy (recovery) and precision (%RSD). For that, method validation was performed according to SANCO guideline 12571/2013. Recovery and RSD were determined intra- and interday by analyzing spiked blank samples with 5 replicates. Each sample was spiked 10–12 h before the extraction and left at 4 °C. Spiked samples were extracted, purified using the homemade SPE, and then analyzed using the same UPLC–MS/MS conditions as described above. RSDs at 3 spiked levels on the same day and 5 consecutive days were used for evaluation of the intra- and interday precision, respectively. The concentrations of mycotoxins in the spiked samples were measured using the matrix-matched calibration curves, and the recovery was calculated as the percentage of the measured analyte concentration divided by the spiked concentration. LOQ was based on a signal-to-noise ratio of 10 or more observed in a sample spiked at 0.5–25.0 μg L^−1^ showing a lower response. Linearity and matrix effects were calculated using solvent- and matrix-matched calibrations in triplicate at 7 concentration levels ranging from LOQ to 200 LOQ. Matrix effect (%) was calculated according to the formula (Am/As) × 100, in which As is the slope of the standard calibration line and Am is the slope of the matrix-matched calibration line.

### 4.8. Statistical Analysis

All data in this study were analyzed using the Statistical Package for Social Science (SPSS) 15.0 for Windows (SPSS Inc., Chicago, IL, USA), and the level of statistical significance was set at *p* ≤ 0.05.

## Figures and Tables

**Table 1:** Accuracy and precision of the analytical method for determining mycotoxins in corn and wheat spiked at three different concentration levels.

Mycotoxins	LOQ (μg L^−1^)	Spiked Levels	Corn			Wheat		
			Recovery (%)	Intraday RSD (%)	Interday RSD (%)	Recovery (%)	Intraday RSD (%)	Interday RSD (%)
DON	10	LOQ	93.4	8.2	9.6	94.7	4.1	6.3
		2LOQ	103.6	2.7	3.2	96.5	1.3	3.1
		10LOQ	101.9	4.1	6.4	98.3	0.8	2.2
NIV	25	LOQ	90.7	5.4	6.8	92.3	2.9	4.3
		2LOQ	99.4	4.8	5.4	99.3	4.4	4.8
		10LOQ	90.7	1.1	2.5	97.2	2.7	3.5
AFB_1_	0.5	LOQ	78.5	3.9	5.6	85.1	2.6	4.2
		2LOQ	80.5	1.7	3.4	82.4	4.4	6.5
		10LOQ	85.6	4.1	5.4	86.9	2.9	4.9
AFB_2_	0.5	LOQ	82.8	2.9	4.8	78.0	5.1	10.7
		2LOQ	87.5	1.8	3.6	80.3	4.6	9.2
		10LOQ	92.8	2.2	4.9	81.9	7.2	8.2
AFG_1_	0.5	LOQ	90.9	3.5	5.6	97.0	3.9	8.1
		2LOQ	87.8	7.1	9.8	87.8	4.2	6.5
		10LOQ	92.7	3.7	6.7	94.8	5.4	6.9
AFG_2_	0.5	LOQ	90.8	3.1	4.2	95.9	3.3	4.2
		2LOQ	103.6	2.8	3.5	90.5	4.0	4.8
		10LOQ	99.7	2.4	3.7	100.9	5.0	6.1
15-AcDON	10	LOQ	91.8	2.8	4.3	100.1	2.0	3.1
		2LOQ	103.3	3.8	4.8	95.3	2.3	3.3
		10LOQ	98.9	4.8	5.7	97.9	0.8	2.1
3-AcDON	10	LOQ	89.2	3.7	4.8	100.0	2.0	3.2
		2LOQ	105.1	5.8	7.1	106.8	3.6	4.5
		10LOQ	93.8	3.9	5.1	100.4	0.4	1.8
FUS-X	10	LOQ	97.1	5.8	7.2	92.8	2.9	4.3
		2LOQ	96.8	2.3	3.4	86.6	3.9	5.8
		10LOQ	84.1	3.2	4.0	101.5	1.1	3.7
DAS	10	LOQ	100.5	3.5	4.2	75.6	7.8	10.4
		2LOQ	84.6	2.3	4.3	93.3	2.0	4.7
		10LOQ	86.8	6.0	8.7	93.2	3.1	6.5
T-2	0.5	LOQ	79.8	4.4	9.6	80.8	4.7	9.4
		2LOQ	104.3	6.0	6.6	104.8	3.3	4.3
		10LOQ	84.9	4.9	7.3	98.8	4.6	5.8
HT-2	10	LOQ	81.4	6.8	8.9	82.2	3.3	5.7
		2LOQ	99.4	6.3	7.5	99.3	2.9	5.3
		10LOQ	89.2	3.2	6.7	96.2	3.7	6.8
NEO	25	LOQ	88.0	7.2	8.9	97.6	4.7	5.9
		2LOQ	101.0	1.9	2.5	97.9	6.0	7.6
		10LOQ	92.3	2.9	4.1	97.9	2.2	3.6
OTA	0.5	LOQ	83.2	2.9	5.4	105.5	3.0	4.2
		2LOQ	110.3	4.4	4.8	108.3	4.9	5.4
		10LOQ	82.0	4.9	6.3	92.0	5.6	6.4
OTB	0.5	LOQ	100.7	2.7	3.9	99.6	3.4	4.8
		2LOQ	101.9	3.6	4.7	100.5	3.5	3.8
		10LOQ	92.2	3.9	5.1	93.5	3.6	4.1
ZEN	0.5	LOQ	96.5	2.2	3.2	93.1	3.0	4.1
		2LOQ	98.5	3.6	4.8	102.0	3.6	4.5
		10LOQ	95.3	3.8	4.6	96.7	2.0	2.8
ZAN	10	LOQ	108.1	5.0	5.6	99.6	4.6	6.2
		2LOQ	106.6	4.1	4.9	103.8	3.2	5.4
		10LOQ	105.4	2.9	3.5	95.7	3.9	4.8
β-ZOL	10	LOQ	102.2	2.8	4.1	99.0	3.1	3.8
		2LOQ	103.0	3.4	4.9	94.0	0.3	1.8
		10LOQ	101.7	1.9	3.5	92.5	3.2	4.1
α-ZOL	10	LOQ	93.8	3.1	5.3	81.2	6.1	9.5
		2LOQ	96.8	1.8	2.4	88.0	4.5	6.3
		10LOQ	96.5	2.0	3.1	89.7	3.5	5.2
α-ZAL	10	LOQ	91.8	2.6	4.2	89.5	5.2	7.8
		2LOQ	93.8	1.6	3.2	96.2	6.3	8.2
		10LOQ	104.6	8.0	7.6	91.8	1.5	4.3
β-ZAL	10	LOQ	91.0	7.9	9.2	94.3	3.1	6.2
		2LOQ	99.1	5.2	7.5	84.6	8.1	9.8
		10LOQ	106.3	3.0	5.4	95.8	3.4	5.2

For each concentration level, mean recovery and relative standard deviation (RSD) were calculated on *n* = 5. LOQ, limit of quantification; DON, deoxynivalenol; NIV, nivalenol; AFB_1_, AFB_2_, AFG_1_, AFG_2_, aflatoxins; 15-AcDON, 15-acetyldeoxynivalenol; 3-AcDON, 3-acetyldeoxynivalenol; FUS-X, fusarenon X; DAS, diacetoxyscirpenol; OTA, ochratoxin A; OTB, ochratoxin B; T-2, T-2 toxin; HT-2, HT-2 toxin; NEO, neosolaniol; ZEN, zearalenone; α-ZOL, α-zearalenol; β-ZOL, β-zearalenol; ZAN, zearalanone; α-ZAL, α-zearalanol; β-ZAL, β-zearalanol.

**Table 2:** Mycotoxins detected in raw samples of corn and wheat using the homemade one-step cleanup procedure with ultraperformance liquid chromatography–tandem mass spectrometry (UPLC–MS/MS) analysis.

Sample (*n*)	Mycotoxin	Positives (*n*)	Occurrence (%)	Mean (μg kg^−1^)	Range (μg kg^−1^)
Corn (59)	AFB_1_	19	32.2	2.18	0.5–26
	ZEN	59	100	69.3	27.5–525
	DON	7	11.9	410.1	65.4–5100
Wheat (53)	ZEN	13	24.5	50.7	26.8–602
	DON	27	50.9	557.5	19.0–4050
	3-AcDON	11	20.8	68.3	13.3–270
	15-AcDON	12	22.6	49.2	10.1–141

Mean concentration of each mycotoxin was calculated on *n* = 3.

## Supplementary Materials

The following are available online at http://www.mdpi.com/2072-6651/10/10/409/s1, Figure S1: UPLC–MS/MS chromatograms of 21 targeted mycotoxins in a spiked (10-times LOQ) solvent sample, Figure S2: Effects of three MWCNT sorbents on matrix effect (%) of mycotoxins in corn. Vertical bar represents ± standard error (*n* = 3), Table S1: Optimized multiple-reaction monitoring (MRM) parameters for the 21 targeted mycotoxins.

## Abbreviations

The following abbreviations are used in this manuscript:

15-AcDON	15-acetyldeoxynivalenol
3-AcDON	3-acetyldeoxynivalenol
AFB_1_	aflatoxin B1
AFB_2_	aflatoxin B2
AFG_1_	aflatoxin G1
AFG_2_	aflatoxin G2
DAS	diacetoxyscirpenol
DON	deoxynivalenol
ESI^−^	negative mode of electrospray ionization
ESI^+^	positive mode of electrospray ionization
FUS-X	fusarenon X
HLB	hydrophilic–lipophilic balanced
HPLC–MS/MS	high-performance liquid chromatography–tandem mass spectrometry
IAC	immunoaffinity chromatography
LOQ	limit of quantification
MCX	mixed-mode cationic exchange
MFC	multifunction cleanup columns
MRM	multiple-reaction monitoring
MWCNT	multiwalled carbon nanotube
MWCNT–COOH	carboxylic MWCNT
MWCNT–OH	hydroxyl MWCNT
NEO	neosolaniol
NH_2_	amino-propyl
NH_4_Ac	ammonium acetate
NIV	nivalenol
OTA	ochratoxin A
OTB	ochratoxin B
QuEChERS	quick, easy, cheap, effective, rugged, safe
RSD	relative standard deviation
SPE	solid-phase extraction
SPSS	Statistical Package for Social Science
UPLC–MS/MS	ultraperformance liquid chromatography–tandem mass spectrometry
ZAN	zearalanone
ZEN	zearalenone
α-ZAL	α-zearalanol
α-ZOL	α-zearalenol
β-ZAL	β-zearalanol
β-ZOL	β-zearalenol
