# Supplementary material for: Multiwalled Carbon Nanotube for One-Step Cleanup of 21 Mycotoxins in Corn and Wheat Prior to Ultraperformance Liquid Chromatography–Tandem Mass Spectrometry Analysis
Source: Toxins (Basel). 2018 Oct 10;10(10):409. doi: 10.3390/toxins10100409 (PMC6215200; doi:10.3390/toxins10100409)
Supplement: Supplementary file 1 [file toxins-10-00409-s001.pdf]

Supplementary Materials: Multiwalled Carbon Nanotube for One-Step Cleanup of 21 Mycotoxins in Corn and Wheat Prior to Ultraperformance Liquid Chromatography–Tandem Mass Spectrometry Analysis

Dongmei Jiang, Dizhe Wei, Liuqing Wang, Shuai Ma, Yuanfang Du and Meng Wang

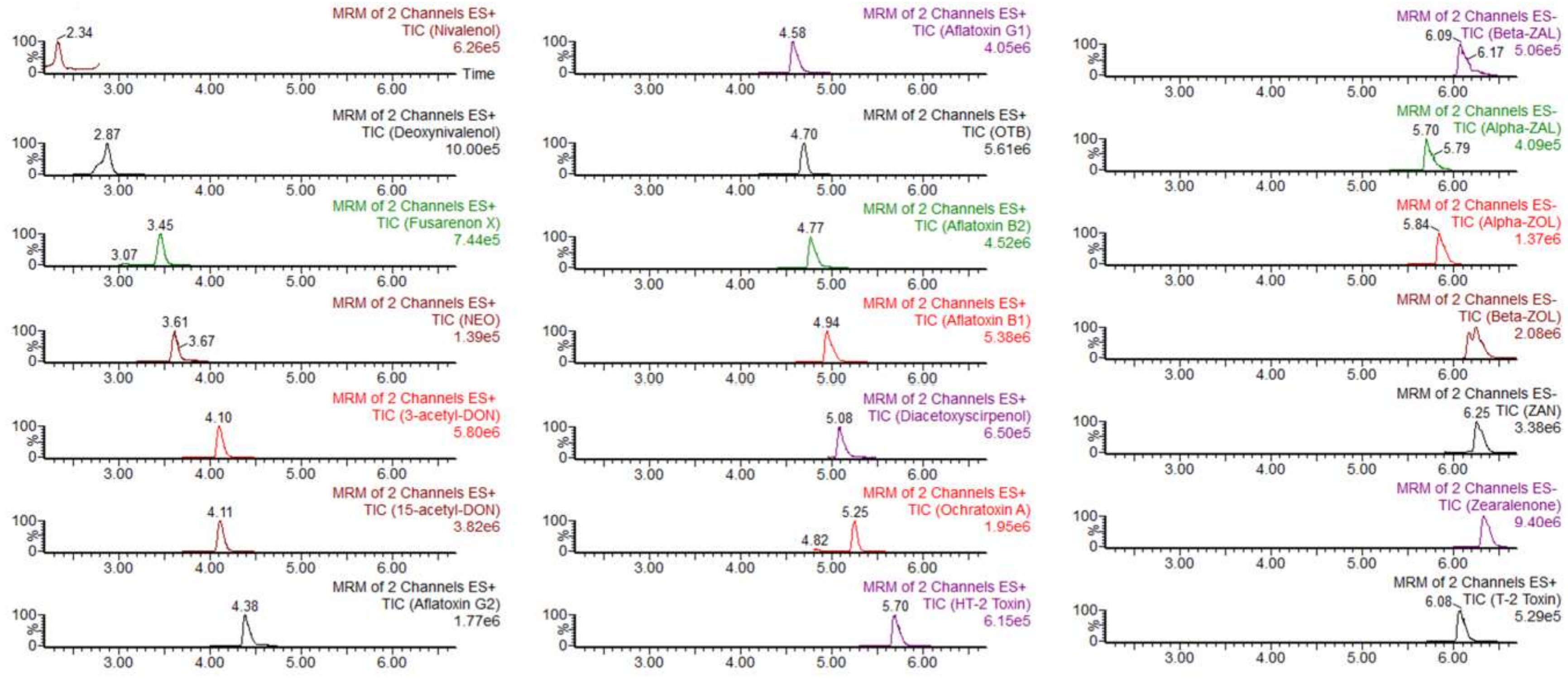

Figure S1. UPLC–MS/MS chromatograms of 21 targeted mycotoxins in a spiked (10 times LOQ) solvent sample.

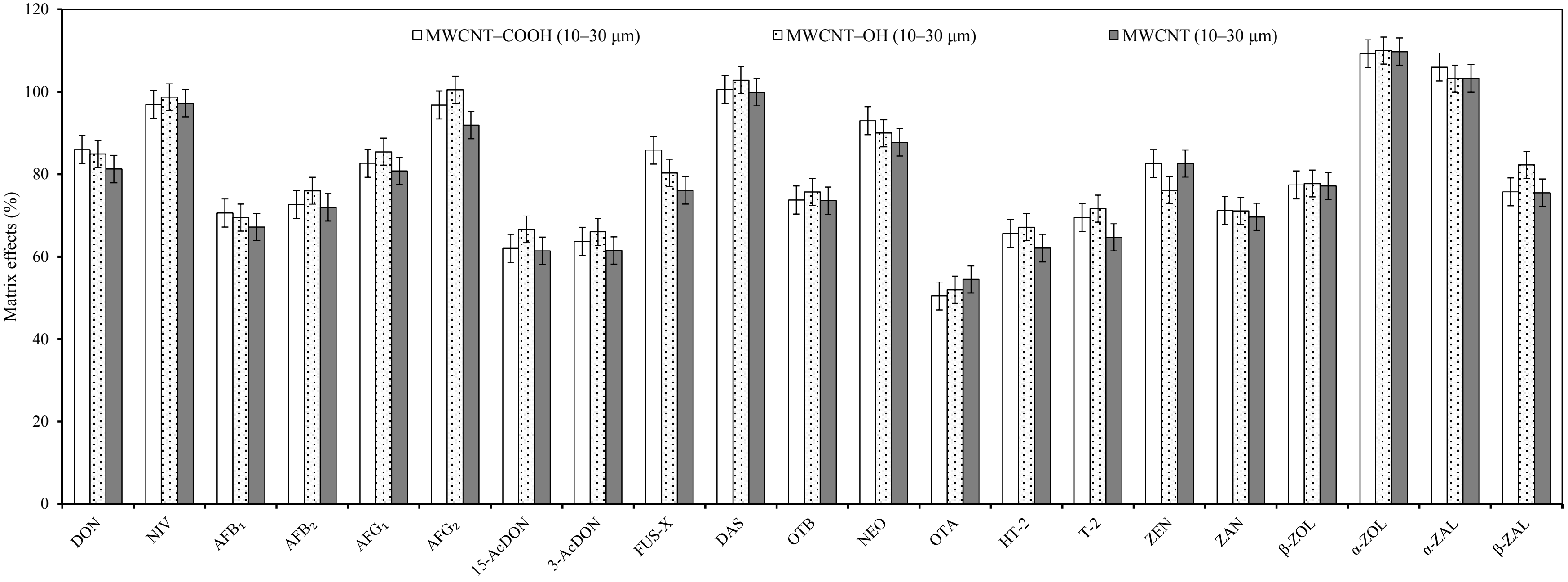

Figure S2. Effects of three MWCNT sorbents on matrix effect (%) of mycotoxins in corn. Vertical bar represents ± standard error (*n* = 3).

Table S1. Optimized multiple reactions monitoring (MRM) parameters for the 21 targeted mycotoxins.

**Mycotoxins**	**Ionization Mode**	**Precursor Ions (*m*/*z*)**	**Quantitative Ions (*m*/*z*)**	**Collision Energy (eV)**	**Qualitative Ions (*m*/*z*)**	**Collision Energy (eV)**	**Cone Voltage (eV)**
OTA	ESI^+	404.2	239.1	19	358.1	14	30
OTB	ESI^+	370.2	205.2	22	187.1	36	32
DON	ESI^+	297.1	249.2	10	102.0	15	28
NIV	ESI^+	313.2	175.1	20	295.1	8	30
NEO	ESI^+	383.1	245.0	10	305.1	10	55
FUS-X	ESI^+	355.2	247.1	15	229.1	10	28
3-AcDON	ESI^+	339.2	231.1	14	203.0	12	28
15-AcDON	ESI^+	339.2	261.1	10	137.0	20	30
DAS	ESI^+	367.2	307.2	10	289.1	12	20
HT-2	ESI^+	442.2	263.1	20	105.1	37	25
T-2	ESI^+	484.2	305.1	8	245.1	9	30
AFB_1	ESI^+	313.2	241.1	36	285.1	25	30
AFB_2	ESI^+	315.2	287.1	26	259.1	30	40
AFG_1	ESI^+	329.2	243.1	25	283.1	25	35
AFG_2	ESI^+	331.2	245.1	30	257.1	30	40
ZEN	ESI^-	317.2	175.4	24	131.1	25	30
ZAN	ESI^-	319.0	205.0	24	163.5	26	30
α-ZOL	ESI^-	319.2	130.0	34	161.0	30	30
β-ZOL	ESI^-	319.2	130.0	34	161.0	30	30
α-ZAL	ESI^-	321.0	161.0	28	205.0	24	30
β-ZAL	ESI^-	321.0	161.0	28	205.0	24	30
